# Biomimetic reduced graphene oxide coated collagen scaffold for in situ bone regeneration

**DOI:** 10.1038/s41598-021-96271-1

**Published:** 2021-08-18

**Authors:** Sajad Bahrami, Nafiseh Baheiraei, Mostafa Shahrezaee

**Affiliations:** 1 Department of Orthopedic Surgery, AJA University of Medical Sciences, Tehran, Iran; 2 Tissue Engineering and Applied Cell Sciences Division, Department of Anatomical Sciences, Faculty of Medical Sciences, Tarbiat Modares University, Tehran, Iran

**Keywords:** Biotechnology, Stem cells, Diseases, Health care, Medical research, Chemistry, Engineering, Materials science, Nanoscience and technology

## Abstract

A variety of bone-related diseases and injures and limitations of traditional regeneration methods require new tissue substitutes. Tissue engineering and regeneration combined with nanomedicine can provide different natural or synthetic and combined scaffolds with bone mimicking properties for implantation in the injured area. In this study, we synthesized collagen (Col) and reduced graphene oxide coated collagen (Col-rGO) scaffolds, and we evaluated their in vitro and in vivo effects on bone tissue repair. Col and Col-rGO scaffolds were synthesized by chemical crosslinking and freeze-drying methods. The surface topography, and the mechanical and chemical properties of scaffolds were characterized, showing three-dimensional (3D) porous scaffolds and successful coating of rGO on Col. The rGO coating enhanced the mechanical strength of Col-rGO scaffolds to a greater extent than Col scaffolds by 2.8 times. Furthermore, Col-rGO scaffolds confirmed that graphene addition induced no cytotoxic effects and enhanced the viability and proliferation of human bone marrow-derived mesenchymal stem cells (hBMSCs) with 3D adherence and expansion. Finally, scaffold implantation into rabbit cranial bone defects for 12 weeks showed increased bone formation, confirmed by Hematoxylin–Eosin (H&E) and alizarin red staining. Overall, the study showed that rGO coating improves Col scaffold properties and could be a promising implant for bone injuries.

## Introduction

Bone tissue has a remarkable self-healing capability for small defects. However, large defects resulting from osteoporosis, tumors, traumatic fractures, and infections cannot regenerate spontaneously, resulting in fractures and deformities^1^. Traditionally, allografts and autografts are used as a common treatment method for bone defects; about 2.2 million grafts occur annually worldwide. Autografts are the most prevalent treatment method, but they are limited by donor site morbidity, high failure rates (up to 50%) and limited graft supply^1,2^. Also, metallic fixtures that are used concurrently with grafts for bone fragments stabilization cause discomfort, infections, long-term complications and repeated surgeries in patients, especially in children^1^. In recent years, new methods have been introduced for fabricating less problematic bone tissue grafts. In this regard, bone tissue engineering possesses great potential for regeneration purposes.

Tissue engineering introduces structural and functional alternatives called scaffold for human tissue imitation. Scaffolds are natural and/or synthetic materials that are implanted into tissue defects. They can imitate the natural three-dimensional (3D) structure of human tissue better than traditional cell culture two-dimensional (2D) plates. Scaffolds are 3D porous structures with similar physicochemical properties with the extracellular matrix (ECM) of the target tissue. In general, scaffolds are biocompatible and biodegradables, and they provide appropriate physical, mechanical, and biochemical support for cell growth and differentiation^3,4^. Optimal bone tissue engineering scaffolds present different structural, biological, and physicochemical characteristics for bone tissue. Bone tissue scaffolds should have 3D and porous structures for in vivo like cell growth and connections, as well as for oxygen/nutrient diffusion. Appropriate biocompatibility and biodegradability provide additional properties that support biological compatibility. Mechanical strength, osteoinductivity and osteoconductivity properties could also be useful for more relevant results and practical clinical applications^5–7^.

In human tissues, cells are surrounded by a microenvironment that includes ECM and other cells. The microenvironment applies a wide range of stimuli, including macro- to nano-scale, chemical, mechanical, and biologic factors that affect cell/tissue fate^7,8^. Bone ECM includes 30% collagen (Col) fibrils and 70% calcium phosphate crystals. Col is the most abundant protein in body tissues and is widely used in biomaterial applications. Col exists in the bone matrix with four different types. Type I makes up 97% of Col part^1,6^. Col type I is a heterotrimer with about 1000 amino acids and a length of 300 nm^9^. Col scaffolds have been widely used for bone tissue engineering due to their abundance, biocompatibility, biodegradability, high porosity, hydrophilicity and low antigenicity^6,9^. For example, Sheyn et al.^10^ used biodegradable Col scaffolds cultured with mesenchymal stem cells to repair radius bone defects. µCT results showed complete bone repair after 8 weeks. High bone density was shown in addition to increased osteogenic markers (BSP and OC). Arakawa et al. fabricated photocrosslinkable methacrylated glycol chitosan (MeGC) and Col hydrogel and showed enhanced bone marrow-derived mesenchymal stem cell (BMMSC) attachment, spreading, proliferation, and osteogenic differentiation^11^.

On the other hand, natural materials encounter limitations, particularly for in vivo applications, that mostly are physicochemical limitations. Novel tissue engineering strategies could overcome these limitations. Bone tissue scaffolds need to have sufficient mechanical properties. Col suffers from weak mechanical properties, causing limited applications^6,9^. Different Col composites with bioceramic components like hydroxyapatite, polymer components like silk fibroin and nanomaterials have been used in bone tissue engineering studies^9^. Combinations of Col with synthetic materials could help strength enhancement and fabrication of in vivo imitating bone scaffolds. For example, in our previous study, a Col/β‐tricalcium phosphate bone graft improved the Col scaffolds mechanical and biological properties and differentiation of BMMSCs into osteoblasts^12^. It has been found that Bioglass 45S5 (BG) incorporated methacrylated Col 3D printed constructs allow improved stability, reduced swelling of Col hydrogels, enhanced alkaline phosphatase activity of stem cells, and cell-mediated calcium deposition^13^.

In this regard, carbon nanomaterials like graphene derivatives could be advantageous. Graphene-based nanoparticles crosslinks have been used to enhance the mechanical properties of Col^14–16^. For example, alginate-chitosan-Col based composite scaffolds consisting of graphene oxide (GO) fabricated by a chemical crosslinking method increased mechanical properties compared to a non-crosslinked method and a method without GO counterparts^17^. Graphene is the most popular carbon nanomaterial and consists of one atomic layer of sp^2^ hybridized carbon atoms. GO is a graphene layer decorated with oxygen-containing functionalities that are reduced in reduced graphene oxide (rGO). Graphene has a large surface area (~ 2600 m^2^ g^−1^), high thermal and electrical conductivity, and significant mechanical properties; it is known as the thinnest, strongest, and stiffest material ever found^1,18^. The physical, chemical, and mechanical properties of graphene derivatives make them ideal for bone regeneration research. Large surface area, biocompatibility, easy handling, tunable mechanical and electrical characteristics make graphene a promising substrate for different cell/stem cell culture and bone studies. Moreover, easy chemical modification of graphene’s surface allows cell adhesion and control over their proliferation and differentiation into osteogenic lineages^1,18^. Graphene also provides mechanical stiffness, electrical conductivity, and chemical modifications for bone scaffolds. Graphene derivatives (whether used alone or composite with other materials) have been extensively studied in bone repair research works. A study on periodontal ligament stem cells culture on 2D and 3D graphene substrates showed enhanced osteogenic differentiation after 28 days. Mouse mesenchymal stem cells cultured on chitosan–gelatin-GO scaffold promoted differentiation into osteoblast in vitro^19^. In vivo studies of the structure showed increased Col deposition and accelerated bridging in rat tibial bone defect. Wua et al.^20^ reported that the osteoinductive properties of graphene enhanced cell adhesion and proliferation in poly(lactic-co-glycolic acid) (PLGA) films cultured with BMMSCs. Better in vivo guiding bone regeneration showed by graphene-PLGA in defective implants than the PLGA group. They claimed that graphene increased osteogenic differentiation and bone regeneration through the PI3K/Akt/GSK-3β/β-catenin signal circuit. Graphene incorporation into various forms of Col has been used for the regeneration of different tissues, such as pegylated GO-Col hybrid scaffold for diabetic wound repair^21^, 3D rGO-Col for neural differentiation^22^, GO-alginate-chitosan-Col for in vitro bone tissue engineering^17^ and Col functionalized with GO for rat cranial defect repair^23^.

In this study, we synthesized Col and rGO-coated Col (rGO-Col) scaffolds by chemical crosslinking and freeze-drying methods. The surface topography and mechanical and chemical properties of rGO-Col scaffolds were characterized, and the results were compared with an uncoated scaffold. Furthermore, the adhesion and proliferation of human bone marrow-derived mesenchymal stem cells (hBMSCs) on scaffolds were studied. Finally, bone formation studies were performed by implanting scaffolds into rabbit cranial bone defects.

## Materials and method

### Scaffold fabrication

Col and Col-Go scaffolds were synthesized via chemical crosslinking and freeze-drying methods according to our previously published protocols^14,24^. Col type I (CCS-1, NZA, Iran) solution (1% acetic acid v/v) was used for Col scaffold synthesis. The Col solution was cast in Teflon molds and was kept at − 20 °C for 5 h and at − 80 °C for 12 h, respectively. Then, it was freeze-dried at − 50 °C for 48 h (freeze drier, ALPH1-2LD, UK). In the next step, crosslinking solution including 1-ethyl-3-(3-dimethylaminopropyl) carbodiimide (EDC; Sigma-Aldrich) and N,hydroxysuccinimide (NHS, Sigma-Aldrich) in ethanol (90% v/v) was added into the samples followed by further freeze-drying for 24 h.

Go solution (400 μg mL^−1^) was used to coat Col scaffolds according to our previously published protocol^14^. Before the coating process, water/GO/EDC (1000:5:4 weight ratio, respectively) was prepared and stirred for 15 min to activate the carboxyl group on the GO plate's surface. The coating process was performed via the immersion of Col scaffolds in a water/GO/EDC solution for 6 h at room temperature. Then, the scaffolds were rinsed with DI water, and the final Col-GO scaffolds were obtained by freeze-drying for 24 h. In preparing Col-rGO scaffold group, a sodium hydrosulfite solution ((Na_2_S_2_O_4_, Sigma-Aldrich) (2%, 3 min) was used to reduce the GO coating. The Col and rGO-Col scaffolds were used for characterizations, and for the in vitro and in vivo studies.

### Scaffold characterization

The porous structure and morphology of the scaffolds were studied by field emission scanning electron microscopy (FESEM) (Tescan, Vega II, Czech). For FESEM studies, the scaffolds were dried and coated with gold. Raman spectroscopy (excitation laser source of 532 nm, Jesco Japan) (only for the rGO-Col group) and a Fourier transform infrared (FT-IR) spectrophotometer (500–3500 cm^−1^, 4.0 cm^−1^ resolution; Equinox-55, Bruker, Madison, Wisconsin, USA) were used for chemical studies of the scaffolds and rGO coating. The liquid displacement method was used to calculate the porosity of Col and Col–rGO scaffolds. Ethanol was used as a liquid medium according to the following equation^25^:

$${\text{Porosity}}\left( \% \right) = \left( {{\text{W}}_{{1}} - {\text{W}}_{{2}} - {\text{W}}_{{\text{S}}} } \right)/\left( {{\text{W}}_{{3}} - {\text{W}}_{{2}} } \right) \times {1}00$$

where W_3_ is the weight of the ethanol dish, W_1_ is the ethanol dish's weight after submersion of each scaffold, W_2_ is the ethanol dish's weight after removing each scaffold, and W_S_ is the weight of each dried scaffold. The mechanical properties of the samples were measured by compressive testing (Instron, Norwood, MA, USA). Prepared samples (height ≈ 7 mm, diameter ≈ 9 mm) were forced at a loading rate of 1 mm min^−1^. Elastic modulus was calculated using the stress–strain curves of compression data.

### Cell culture and cellular evaluations

Cellular experiments were conducted using hBMSCs cells (Royan institute, Iran). In vitro cytotoxicity assays were performed in three different groups: Col, Col-rGO scaffolds and TCP. Stem cells were cultured in the standard culture medium included by Dulbecco's modified Eagle medium (DMEM, Invitrogen), fetal bovine serum (FBS, 10% (v/v), Gibco), and an antibiotic (1% penicillin/streptomycin, Sigma-Aldrich). Cells were incubated in an incubator at 37 °C and 5% CO_2_. Cell passage were performed after 80% confluency and passages three cells were used for cellular studies.

Scaffolds were sterilized before cell culture by ethanol (75%) and UV exposure. Cell viability and proliferation were examined by MTT (3-[4,5-dimethylthiazol-2-yl]-2,5-diphenyltetrazolium bromide) colorimetric assay with hBMSCs culture after 48 and 96 h. For this assay, 10^4^ cells were seeded on Col, Col-rGO scaffolds, and tissue culture plate (TCP) in 96-well culture plates (n = 3/group). The samples were incubated in an incubator at 37 °C and 5% CO_2_. At each time point, the culture medium was removed, and then, the MTT solution (5 mg mL^−1^ in PBS) was added to each sample and incubated for 4 h. The MTT solution was then removed, and 100 µL dimethyl sulfoxide (DMSO; Sigma-Aldrich) was added to each well in order to dissolve the formazone precipitates. At each time point, three samples were used, and TCP was considered as the control group. The optical absorbance was measured using a microplate reader (ELISA reader, BioTek) at a wavelength of 570 nm.

For the morphology assessment, the samples were incubated with culture medium for 24 h in an incubator at 37 °C and 5% CO_2_. hBMSCs were cultured on the scaffolds for 48 h. After that, scaffolds were rinsed with PBS, and cells were fixed by 2.5% glutaraldehyde (1 h). Then, samples were dehydrated using ethanol series (10, 30, 70, 90, and 100%), and cell morphology was studied using FESEM microscopy.

### In vivo experiments

#### Craniofacial bone defect surgery procedures

Before animal surgery, the circular scaffolds (8 mm diameters, circular and 2 mm thickness) were prepared and sterilized by ethanol (75%) and UV exposure. All methods were performed in accordance with the relevant guidelines and regulations. Eight New Zealand white rabbits (average weight of 2–2.5 kg) were used randomly for this experiment (n = 4/group). Animals were treated according to the animal care guidelines (access to food and water, 12 h lighting and 12 h darkness at 20–25 °C) approved by the Ethics Committee of Aja University of Medical Sciences and according to ARRIVE guidelines. The animals were anesthetized with a mixture of ketamine and xylazine intramuscular injection. The surgical site was shaved and scrubbed with povidone-iodine. After preparation, a surgical midline incision of approximately 3 cm was made, and calvaria was exposed after soft tissue and periosteum dissection. Then, two circular calvarial defects (8 mm diameters) were created in each rabbit by a surgical drill under irrigation. The drilled bone was removed, and each scaffold (Col and Col-rGO) was implanted in one defect. The tissue layers were closed by 3-0 absorbable sutures, and the animals were watched for 12 weeks.

### Histological study

The animals were sacrificed 12 weeks after surgery. Specimens were prepared for Hematoxylin–Eosin (H&E) and alizarin red staining assays. The bone specimens, including the scaffold sections and additional surrounding host bone, were removed for analysis. Rabbit bones were decalcified in a 10% EDTA solution (Sigma-Aldrich, USA), followed by being fixed by paraffin and stained with H&E and alizarin red. The sections were taken from different parts of the samples and observed using light microscopy (Leica Microsystems AG, Germany).

### Statistical analysis

All experiments were performed in triplicate biologically independent replicates. Data were analyzed using one-way ANOVA and Tukey’s tests (REST 2009 V2.0.13 software). p values of < 0.05 were considered significant.

## Results and discussion

Different natural or synthetic materials have been used for scaffold fabrication in the field of bone tissue engineering^9,26–29^. Natural materials have shown more appropriate regeneration properties, mostly due to their similarity to mammalian cell ECM. In this regard, Col is the most prevalent protein in human tissues and is extensively used for biomaterial research^9^. Col exists in bone ECM and forms bone cell and tissue structural support, combined with mineral materials like calcium phosphate crystals. Col’s exclusive properties, including availability, biocompatibility, biodegradability, high porosity, and low immunogenicity, have resulted in extensive bone tissue engineering applications^9^. Col provides easy processing and combination with other materials. Col has been used for injecting hydrogels, films and membranes, sponges, and micro/nanospheres in bone regeneration studies^9^.

**Figure 1:**
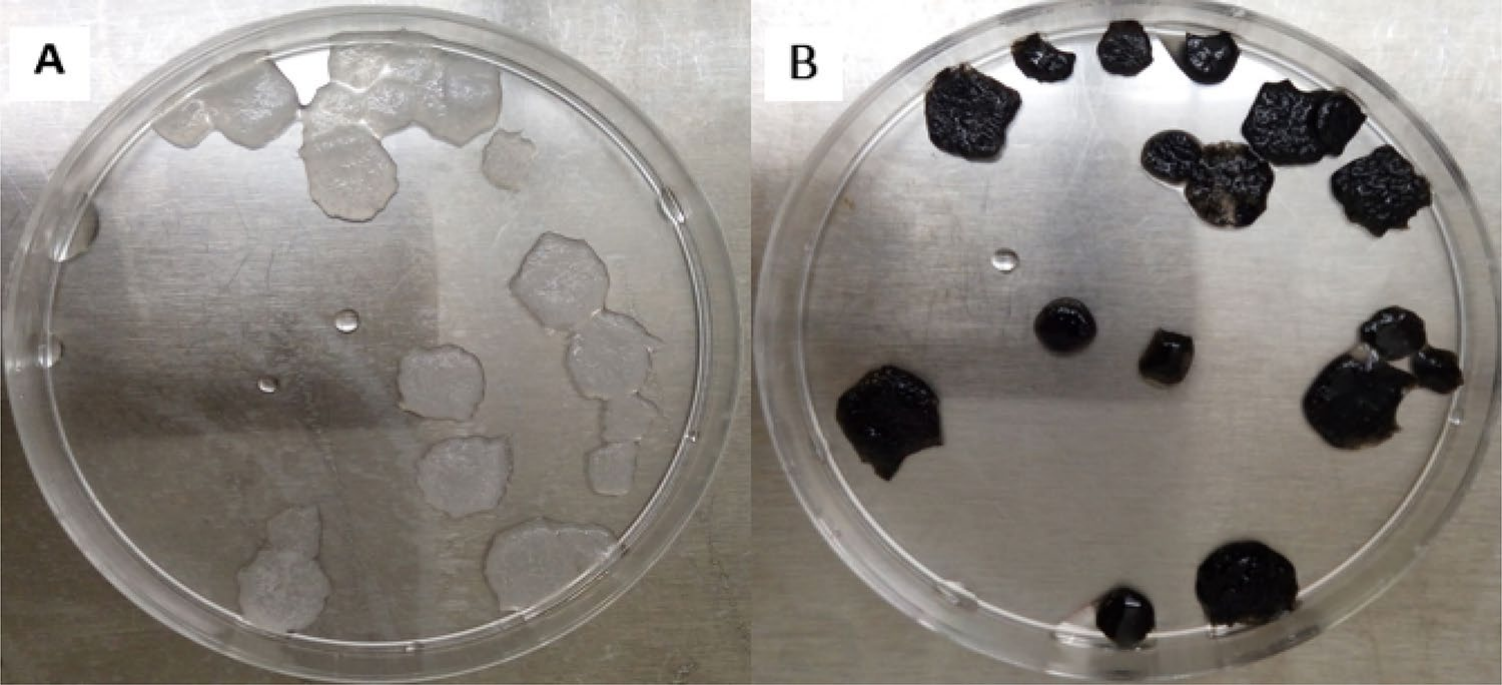
Macroscopic structure of (**A**) Col and (**B**) Col-rGO scaffolds. Graphene coating darkened the scaffolds color without any change in stability.

**Figure 2:**
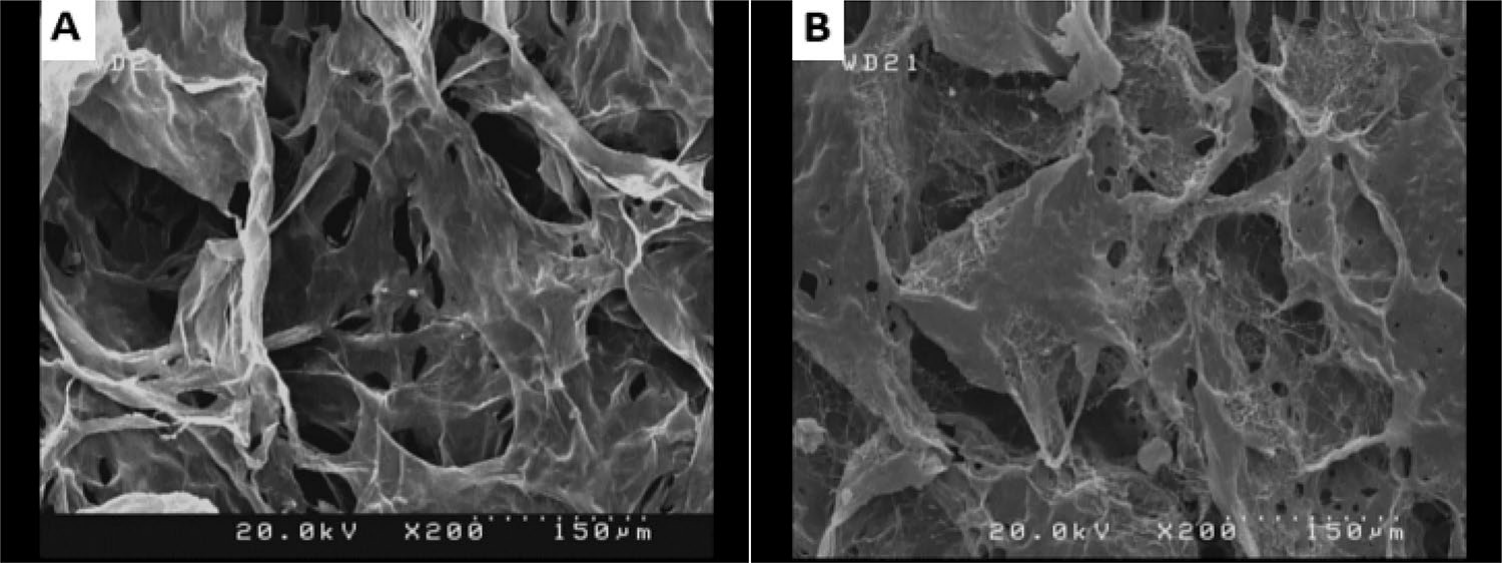
FESEM images of Col (**A**) and Col-rGO (**B**) scaffolds, depicting 3D porous structure.

The macroscopic structure of Col and Col-rGO are shown in Fig. 1A,B, respectively. Graphene coating has darkened the scaffolds color. The structure and stability of the scaffold were not changed after the coating process. Figure 2 depicts morphological and structural characteristics of scaffolds during the synthesis process resulting from FESEM. The results showed 3D porous structures in both scaffolds. The 3D structure of the Col scaffold did not change after the GO coating and reduction process, and a 3D porous framework could be seen (Figs. 1, 2). Bone scaffolds should provide 3D porous structure mimicking an in vivo like bone-forming environment. Col's structure and composition flexibility have resulted in different structure scaffolds, including 3D porous Col sponges and hydrogels. An optimized protocol based on chemical crosslinking and freeze-drying resulted in a 3D porous scaffold (Fig. 2), which is in accordance with our previous studies^14,24^ and that of Liu et al.^30^.

**Figure 3:**
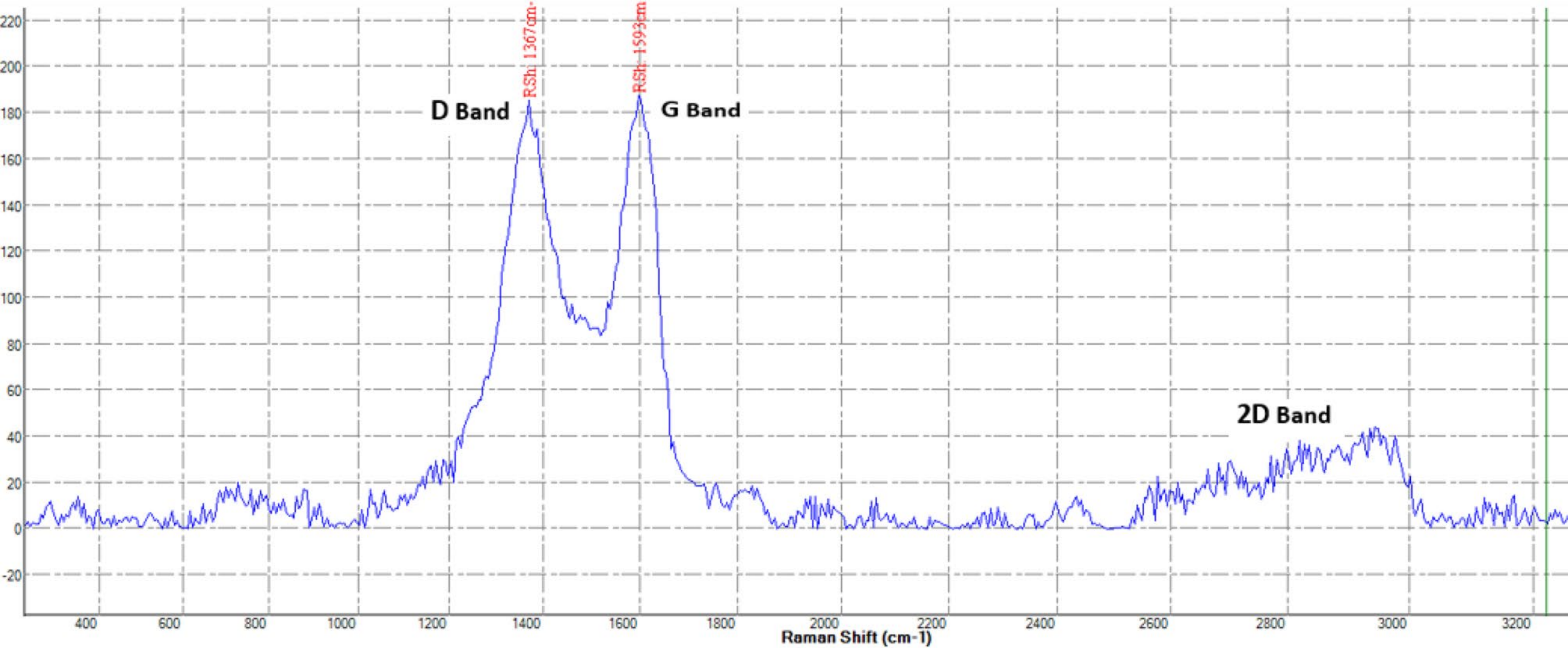
Raman spectrum of the Col-rGO scaffolds showing D, G and 2D bands.

**Figure 4:**
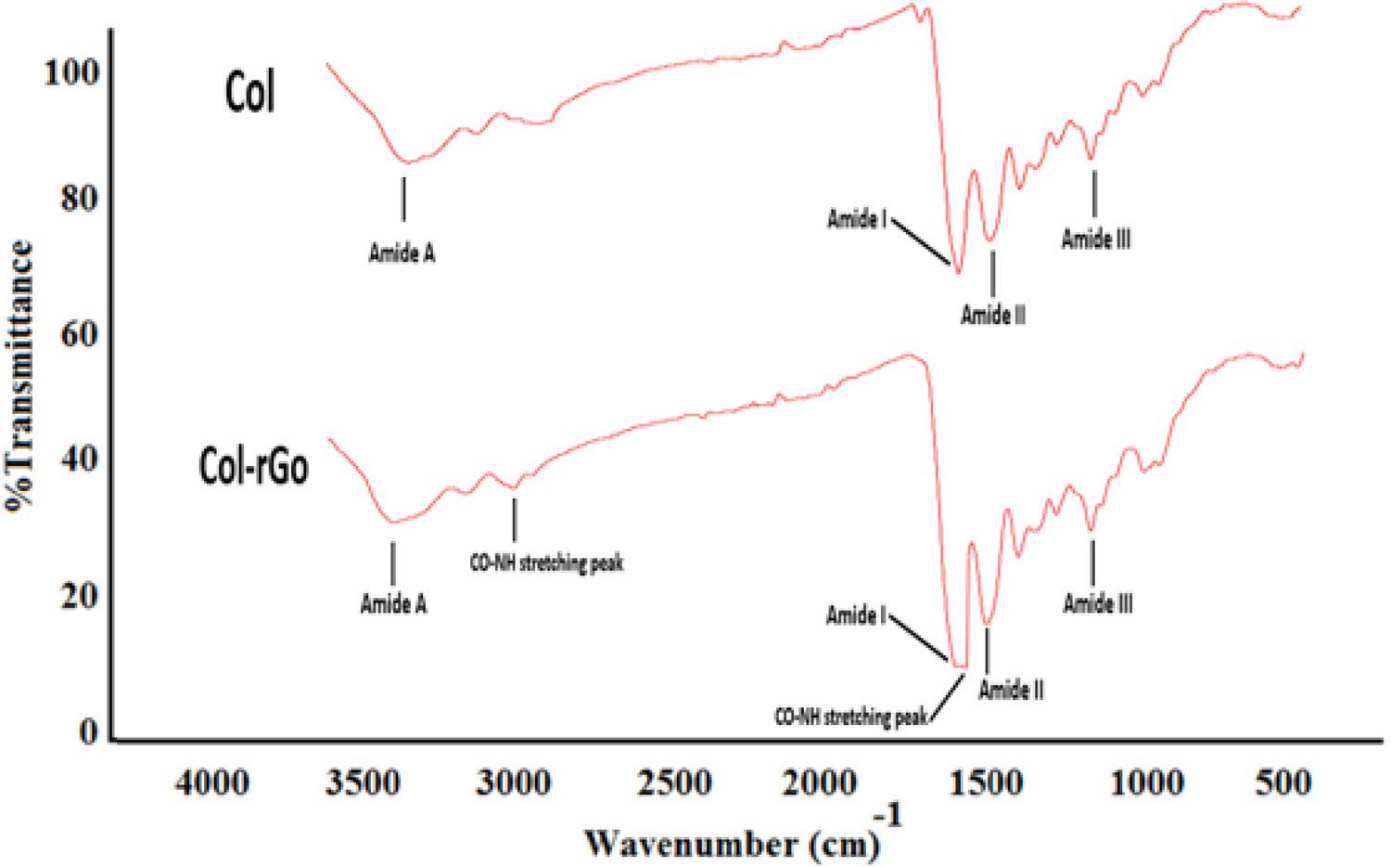
FTIR spectra of the Col and Col–rGO scaffolds, illustrating changes in the functional groups after graphene coating.

Raman spectroscopy was used to detect the typical graphene peaks on Col-rGO scaffolds. Figure 3 shows three main peaks: D (ranging from ~ 1300–1400 cm^−1^), G )ranging from ~ 1500 to 1600 cm^−1^(, and 2D (ranging from ~ 2700 to 2900 cm^−1^) in Col-rGO scaffolds, thus confirming the successful coating of graphene on Col scaffolds. The G/2D ratio was used to determine the number of graphene layers. The ratio showed the coating of multi-layer graphene-coated structures. The FTIR spectra of Col and Col-rGO scaffolds are shown in Fig. 4. The Col scaffold spectra resulted in amide I, II, III, and amide A peaks at 1628, 1535, 1233, and 3274 cm^−1^, respectively^24,31^. The Col-rGO scaffold spectra showed the same amide groups in addition to 1615 and 2929 cm^−1^ peaks, which are related to the CO–NH stretching peak^14,32^. Chemical crosslink bonds Col amine or hydroxyl groups and GO sheet’s oxygen-containing functional groups (carboxyl group (COOH) and alkoxy) (Figs. 3, 4).

The liquid displacement method showed 96.4% porosity for Col and 94% porosity for Col-rGO scaffolds. The porosity difference between scaffolds before and after the rGO coating process was not significant, indicating the fabrication of highly porous scaffolds for cellular studies. Compressive testing was used to determine the effects of the graphene coating on the Col scaffold's mechanical properties. The stress–strain curves of the compression test on Col and Col-rGO scaffolds were used to determine the scaffolds’ elastic modulus and mechanical strength. The results showed a 325 ± 18 kPa of elastic modulus for the Col-rGO scaffold and a 115 ± 16 kPa elastic modulus for the Col samples. Bone is a load-bearing tissue with high strength, and, therefore, bone tissue scaffolds need to have sufficient mechanical properties. It was observed that Col has weak mechanical properties for bone structural support and bone differentiation^9,33^, thus requiring an additional part to enhance scaffold strength. It is also reported that Col's mechanical properties could be enhanced by crosslinking with graphene-based nanoparticles^14–16^. Liu et al.^30^ significantly increased the elastic modulus of Col from 0.2 to 0.34 MPa by adding 0.1% GO. In this study, Col and Col-rGO scaffolds represented 115 ± 16 kPa and 325 ± 18 kPa elastic moduli, respectively, which is similar to our previous study^14^. Graphene nanosheet coatings on the Col structure enhanced the mechanical strength of the scaffold 2.8-fold. Using this scaffold, cells use mechanosensing and differentiate on a substrate similar to native bone tissue. In addition, high-strength scaffold implantation prevents additional scaffold and bone remodeling and damage to the injured area. Also, the limited electrical conductivity of natural biomaterials challenges their practical application. It has been reported that the osteoinductive properties of graphene-incorporated PLGA films enhance the bone differentiation of stem cells and guide bone tissue regeneration^20^. Therefore, the exceptional mechanical and electrical conductivity of rGO which is significantly more electrically conductive than GO on Col scaffolds seems to be helpful in bone repair.

**Figure 5:**
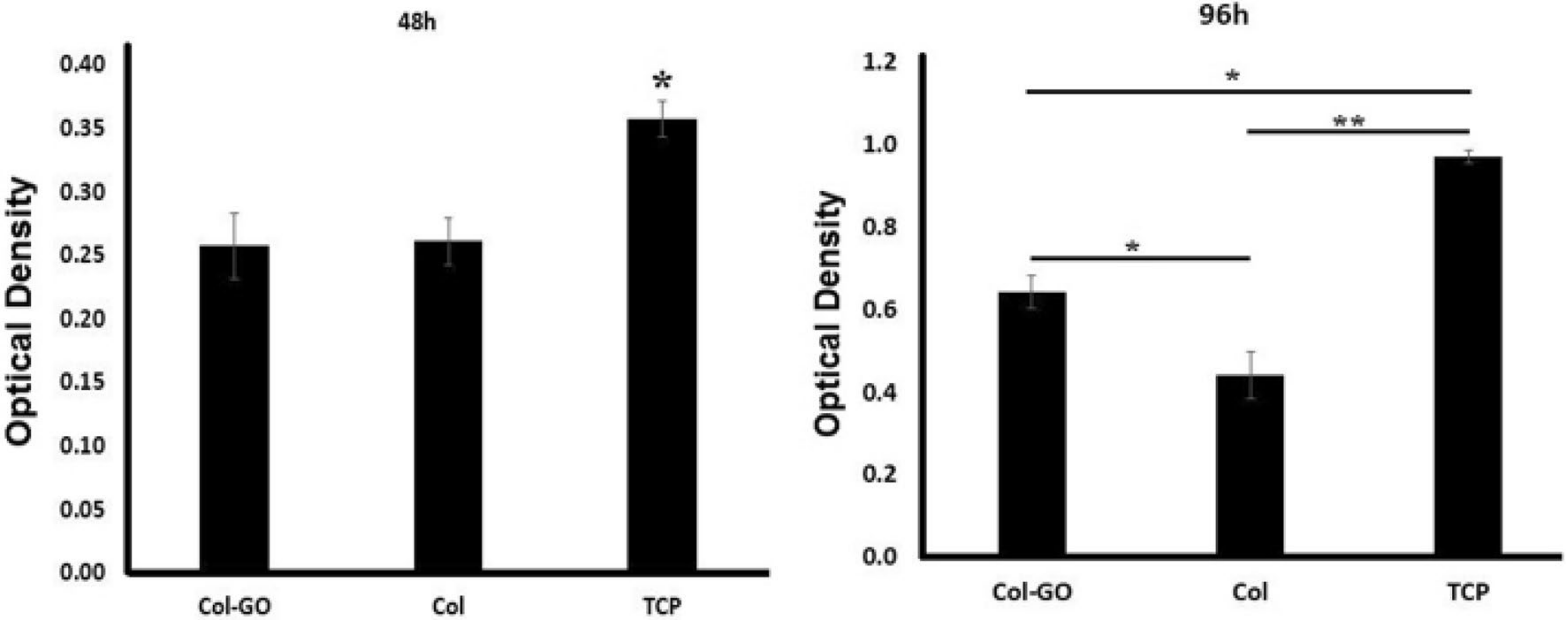
MTT assay results of hBMSCs cultured on Col, Col-rGO scaffolds and TCP group. *(p < 0.05) and **(p < 0.01) indicate significant difference compared with other groups. Data were presented as the average ± standard deviation (n = 3/group).

**Figure 6:**
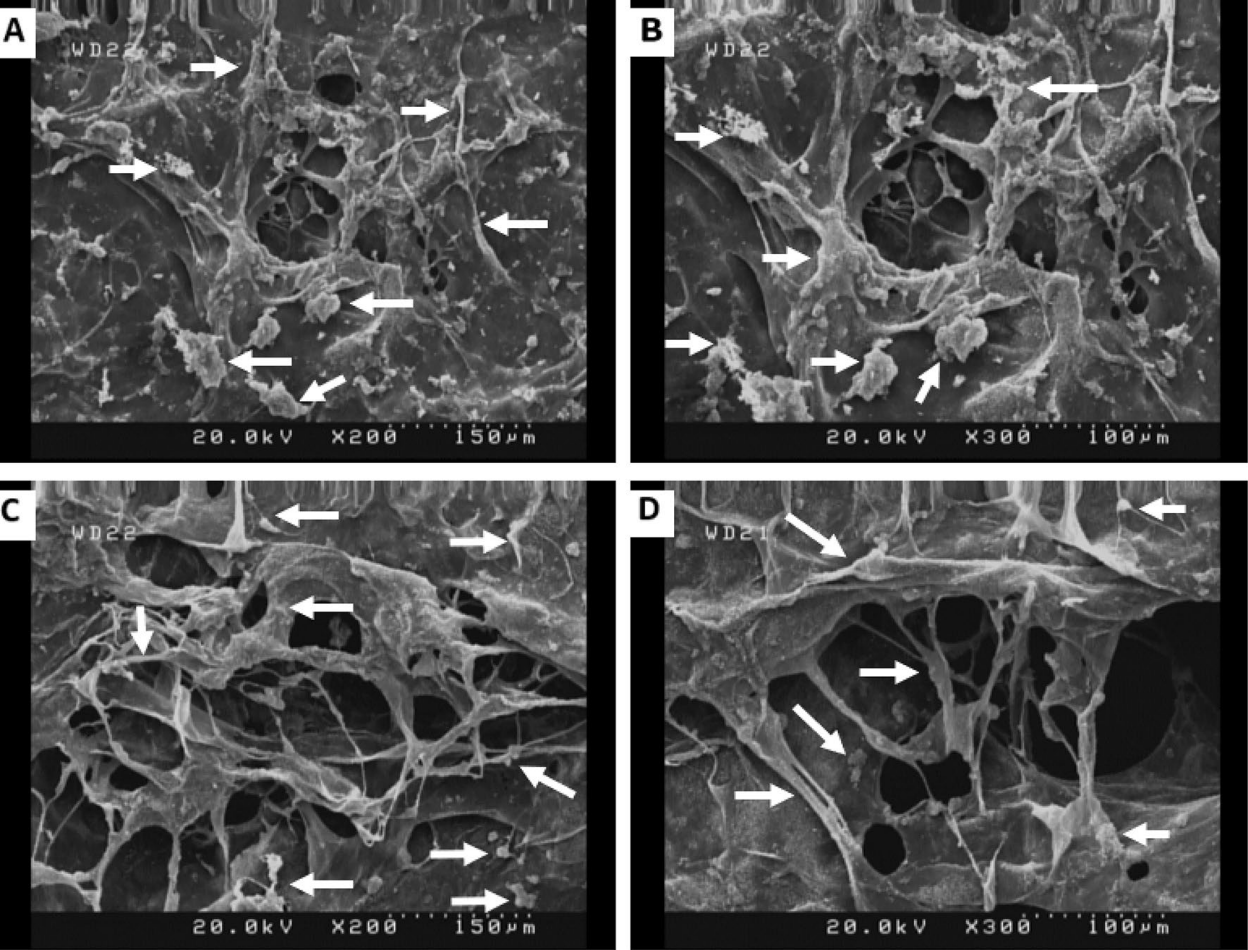
SEM images of hBMSCs seeded on Col (**A**,**B**) and Col-rGO (**C**,**D**) scaffolds after 48 h. Some cells are marked by arrows.

The cytotoxicity of the samples was evaluated using MTT assay. MTT results (Fig. 5) showed more cell viability in Col-rGO scaffolds than the pristine Col, which was significant after 96 h (p < 0.05). These results confirm that addition of graphene did not induce any cytotoxic effects while also enhancing cell viability and proliferation. The adhesion of BMSCs on scaffolds was evaluated by FESEM microscopy. After 48 h of hBMSCs seeding on scaffolds, images were taken (see Fig. 6). Stem cells adhered and grew on both scaffolds. In both groups, the stem cells were attached and expanded on 3D scaffolds and penetrated into pores. 3D adherence and expansion with natural morphology are apparent in Col-rGO scaffolds. Cells used the 3D porous structure of graphene-coated scaffolds for enhanced adhesion, proliferation, and cell–cell contact.

The cytotoxicity of GO and rGO depend on the number of layers, concentration, lateral dimensions, shape, and cell type^12,24^. For example, our previous study on different concentration ranges of rGO from 200 to –800 μg mL^−1^ on Col scaffolds showed that a concentration of 400 μg mL^−1^ allows the most cell viability for HUVECs cells^14^. Another study reported that 0.1% GO–Col aerogels group exhibits better cytocompatibility than 0.05% and 0.2% groups on rat BMSCs^30^. The addition of GO to alginate-chitosan-Col based composite scaffolds did not change the metabolic activity of MC3T3 osteoblast cells. It also provided better support for cell proliferation compared to a GO nonincorporated group^17^. GO addition induced increased osteogenesis and as a result, calcium mineral deposition. The cytotoxicity results of this study on Col-rGO showed more biocompatibility than pristine Col on hBMSCs after 96 h of seeding (Fig. 5). The same results were achieved for cell adhesion (Fig. 6). Despite the adhesion and expansion on both scaffolds, Col-rGO scaffolds showed enhanced adhesion, proliferation, and expansions called cells developing spreading cytoplasmic projections, penetration into pores and cell–cell contact.

Different structural and physicochemical characteristics of Col-rGO scaffolds could be involved in these cellular results. The 3D structure of the Col-rGO scaffold provides a large surface area for hBMSCs stem cells to attach and grow in different directions in an in vivo-like structure. Tissue scaffolds should have a 3D structure and interconnected pores to promote appropriate cell adhesion and interconnection, in addition to nutrients and oxygen transport. Significant porosity of 94% for the Col-rGO scaffold—which is higher than that of the GO–Col scaffold (83%)—synthesized by mixing Col and GO solutions^30^) is sufficient for cell migration and vascularization^34,35^. High porosity facilitates stem cells’ interconnection and medium transport. The creation of a cellular framework could be seen in Col-rGO scaffolds, which is necessary for constructing larger tissues. Moreover, the scaffolds' topological properties provide additional support for cell adhesion, proliferation, and viability. It is confirmed that ripples and wrinkles on graphene nanosheets result in better cell adhesion^25,36,37^. Stiffer scaffolds activate molecules involved in cell adhesion and proliferation^30,38^. It has been reported that high strength, 3D structure and porous graphene foam induce spontaneous osteogenic differentiation for hMSCs^39^. Other surface properties like existing oxygen-containing functional groups such as hydroxyl, carboxyl, epoxy, and free surface π electrons allow hydrogen bonding, π–π interactions, and other surface reactions, thus providing additional surface reaction sites^24,40^. Kolanthai et al.^17^ reported that a negatively charged surface of GO is favored by osteoblast cell adhesion, growth, and proliferation. Surface sites can adsorb serum proteins, such as fibronectin, in culture media and provide a hydrophilic surface, thereby enhancing stem cell adherence, viability, and proliferation. These interactions have been confirmed in other studies using different graphene materials^24,37,41,42^. The abovementioned properties encounter some types of reported graphene cytotoxicity damage, such as ROS production^43,44^, physical damage to the cell membrane by graphene sharp edges^45,46^.

**Figure 7:**
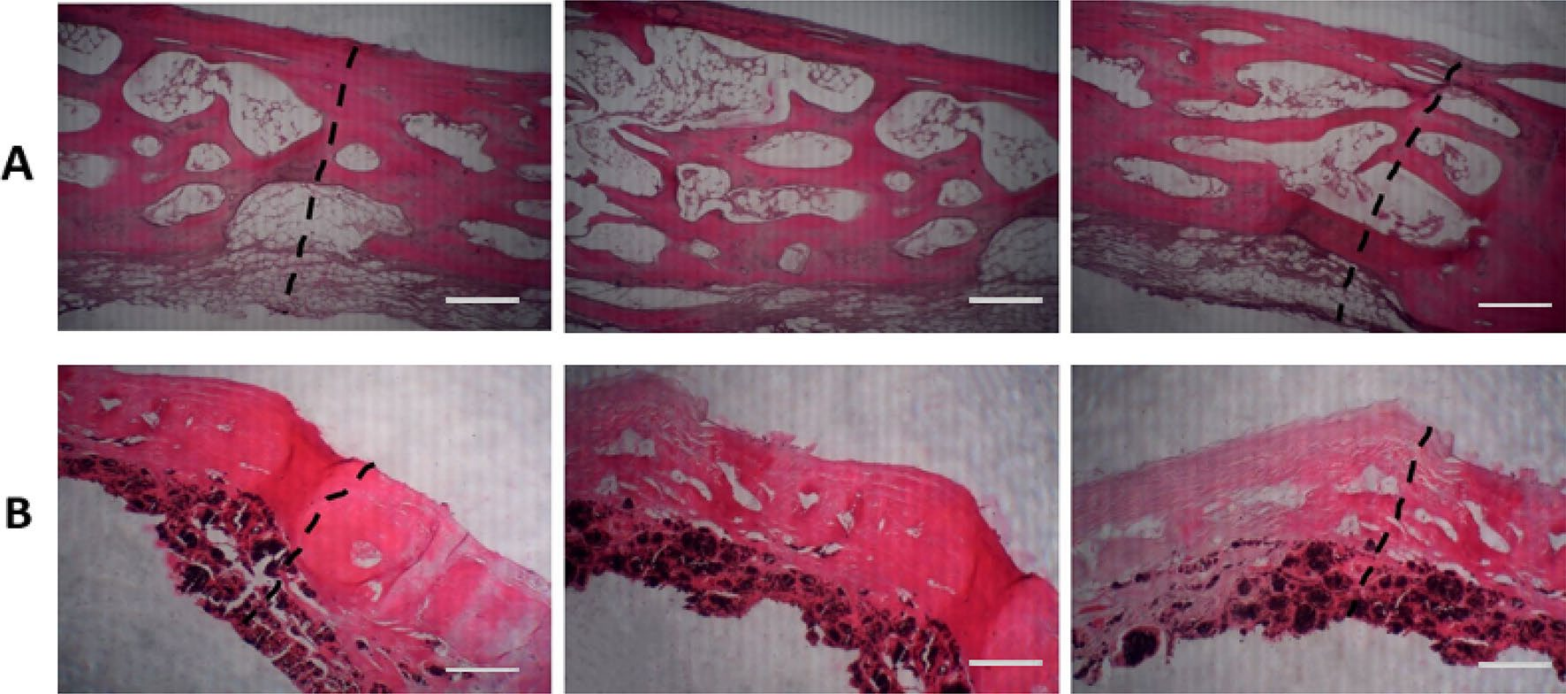
Histological findings after 12 weeks; H&E stain of the bone defects repaired by Col (**A**) and Col-rGO (**B**) scaffolds. Images for each group were illustrated from three different parts of the same location. The dotted line indicates the border between intact bone and scaffolds. Scale bar 100 µm.

**Figure 8:**
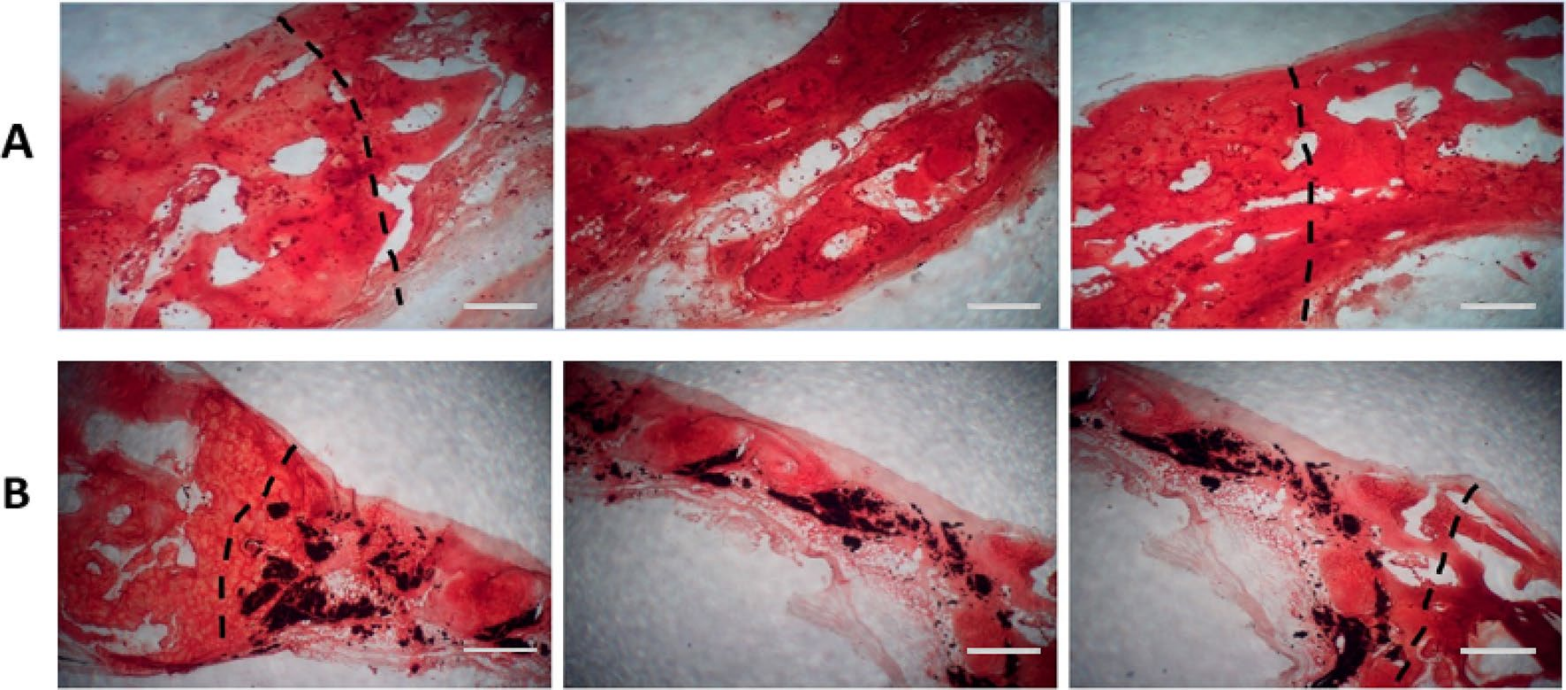
Histological findings after 12 weeks; Alizarin Red stain of the bone defects area repaired by Col (**A**) and Col-rGO (**B**) scaffolds. Images for each group were illustrated from three different parts of the same location. The dotted line indicates the border between intact bone and scaffolds. Scale bar 100 µm.

Histological analysis was used to examine the presence of new bone formation in the defect area. H&E (Fig. 7) and alizarin red (Fig. 8) staining were performed on rabbit cranial defect samples. H&E results showed no necrosis or inflammation for either group. The abovementioned advantages of Col-rGO scaffolds could be involved in animal study results. This theory was tested using an 8-mm critical-sized rabbit cranial defect model. As shown in Fig. 7, H&E results showed that new osteogenesis and cell migration in different regions of the implanted Col-rGO scaffolds exceed those of their Col counterparts. Alizarin red staining was used to determine mineralization and bone formation. The results (Fig. 8) showed that mineralization in central regions of the defect could be seen more clearly for implanted Col-rGO scaffolds than for the Col scaffolds. Similarly, the area surrounding the defects showed more mineralization and new bone formation.

Enhanced in vivo results follow the biological, physicochemical, and topological properties of the Col-rGO scaffolds (e.g., 3D, porous structure, biocompatibility, enhanced cell adhesion, high mechanical strength, and specific surface factors), which are discussed above. The results are in accordance with those of similar in vivo studies. For example, a 0.1% GO–Col aerogel implant into rat cranial defect models showed better bone repair than a Col aerogel^30^. In vivo results of graphene-contained scaffolds indicate the scaffold’s osteogenesis properties without any external ingredients like growth factors or cells. Different studies have used external osteogenesis factors along with scaffolds. For example, researchers have used ECM components, cells, and growth factors along with Col scaffolds to promote osteogenesis^47^. In addition to the discussed reasons, two more advantages of Col–rGO scaffolds for in vivo bone-forming could be related to the angiogenic and antibacterial properties of graphene. The addition of rGO flakes within MSC spheroids upregulated the expression of VEGF growth factors, cell–ECM interaction, and enabled cell signaling cascades^42^. The low levels of ROS production associated with GO incorporation and the activation of phospho-eNOS and phospho-Akt by rGO could be pro-angiogenic signaling factors that induce vascularization^42,48,49^. Govindarajan et al. found that 3D porous Col aerogel exhibits cytocompatibility, as well as and wound healing and angiogenesis effects^50^. Our scaffold fabricated by Col–rGO400 μg mL^−1^ confirmed that VEGF-induced angiogenesis occurs 4 weeks after the introduction of a subcutaneous implant^24^. In addition, graphene materials present antibacterial and antifungal properties^51,52^ that are promising for bone tissue engineering applications. Our previous study confirmed that Col-rGO scaffolds induce antibacterial effects against E. coli, and S. aureus pathogens through the loss of bacterial membrane integrity and the generation of oxidative stress^14^. Col–rGO scaffolds’ antibacterial properties could be advantageous in preventing biofilm and implant infections and subsequent successful practical applications.

## Conclusion

In this study, we fabricated Col and Col-rGO scaffolds via a chemical crosslinking and freeze-drying method. Our findings showed that Col-rGO scaffolds exhibited 3D porous structures with better biochemical properties and mechanical strength than Col scaffolds. Moreover, Col-rGO scaffolds provided better adhesion, viability, and proliferation for hBMSCs cells. The Col-rGO scaffolds exhibited more bone formation than Col scaffolds in in vivo study in rabbit cranial defect models. Overall, the study showed that rGO coating improves Col scaffold properties and could be a promising implant for bone injuries.
